# Context-dependent functions of specific microRNAs in neuronal development

**DOI:** 10.1186/1749-8104-5-25

**Published:** 2010-10-01

**Authors:** Fen-Biao Gao

**Affiliations:** 1 Departments of Neurology and Neurobiology, University of Massachusetts Medical School, Worcester, MA 01605, USA

## Abstract

MicroRNAs (miRNAs) are small noncoding RNAs that regulate multiple developmental processes at the post-transcriptional level. Recent rapid progresses have demonstrated critical roles for a number of miRNAs in neuronal development and function. In particular, miR-9 and miR-124 are specifically expressed in the mammalian nervous system, and their respective nucleotide sequences are 100% identical among many species. Yet, their expression patterns and mRNA targets are less conserved throughout evolution. As a consequence, these miRNAs exhibit diverse context-dependent functions in different aspects of neuronal development, ranging from early neurogenesis and neuronal differentiation to dendritic morphogenesis and synaptic plasticity. Some other neuronal miRNAs also exhibit context-dependent functions in development. Thus, post-transcriptional regulation of spatial and temporal expression levels of protein-coding genes by miRNAs contributes uniquely to the proper development and evolution of the complex nervous system.

## Background

MicroRNAs (miRNAs) are small, noncoding RNAs (21 to 24 nucleotides) that are processed from hairpin structures derived from endogenously transcribed primary miRNAs (pri-miRNAs) [1,2]. As part of Argonaute complexes, these small RNAs regulate gene expression at the post-transcriptional level through imperfect base-paring with specific sequences, located mostly in the 3' UTRs and, in some cases, in the 5' UTRs or the coding regions [3-6]. Each miRNA is predicted to regulate up to hundreds of mRNAs [7]. These miRNA-target interactions often result in mRNA degradation but, under certain circumstances, may also increase the translation of some target mRNAs [6,8,9].

Since the first miRNA was discovered in *Caenorhabditis elegans *in 1993 [10], and the second miRNA along with its evolutionary conservation in 2000 [11,12], hundreds of miRNAs have been identified. miRNAs have been implicated in almost all aspects of cellular processes, including developmental timing, tumorigenesis, immunity, neuronal development, and neurodegeneration [13-18]. These regulatory small RNAs can function as developmental switches or fine-tuning systems to ensure robustness [19,20]. In some other cases, loss of individual miRNAs does not seem to lead to any gross developmental defects but may reveal specific functions under sensitized genetic backgrounds [21].

In the nervous system, recent studies in several model organisms demonstrate critical roles for a number of miRNAs in neuronal development or function. For instance, Lsy-6 and miR-273 are engaged in a feedback loop in specifying the cell fate of two chemosensory neurons in *C. elegans *[22]. miR-7 promotes photoreceptor neuron differentiation through modulating components in the epidermal growth factor receptor signaling pathway in *Drosophila *[23]. In mammals, miR-134 plays a prominent role in regulating dendritic spine morphogenesis through LIM domain kinase 1 (Limk1) [24] and members of the miR-200 family are involved in the terminal differentiation of olfactory precursors [25]. Interestingly, miR-134 also regulates sirtuin 1 (SIRT1)-mediated synaptic plasticity and memory formation [26] and embryonic stem cell differentiation [27], suggesting miRNAs can exert developmental and cellular context-dependent functions. Consistent with this notion, multiple functions of miR-132 have been revealed. miR-132 is regulated by the cAMP response element binding protein (CREB) and in turn affects neurite outgrowth through the Rho family GTPase activating protein p250GAP [28]. miR-132 also modulates the circadian clock located in the suprachiasmatic nucleus [29] as well as antiviral innate immunity in monocytes and primary lymphatic endothelial cells [30]. Moreover, miR-138 is involved in both spine morphogenesis [31] and cardiac patterning [32].

In this review, I will focus on miR-9 and miR-124, two miRNAs that are specifically expressed in the mammalian nervous system. They are highly conserved at the nucleotide sequence level in different species yet exert diverse context-dependent functions through different mRNA targets. Thus, as the most extensively studied neuronal miRNAs, their roles in various aspects of neuronal development in different species will serve as an excellent case study to elucidate the functional conservation and divergence of neuronal miRNAs during evolution.

## miR-9 and miR-124: mammalian brain-specific miRNAs

miR-9 (also known as miR-9a in *Drosophila*) was first identified in *Drosophila *[33] and its authenticity and conservation were confirmed by its identification in mouse brains [34-36]. miR-9 is highly conserved at the nucleotide sequence level from flies to humans but not in *C. elegans*. In *Drosophila *embryos, miR-9 is highly expressed in ectodermal epithelial cells, with little or no expression in the central nervous system [37,38]. In contrast, miR-9 in rodents is specifically expressed in the brain but not other tissues; in the brain, it is broadly expressed in neuronal precursors and also at lower levels in some postmitotic neurons [34-36]. Thus, although this miRNA is highly conserved at the nucleotide level, its tissue-specific expression pattern is not.

In mammals, miR-9 is processed from three precursors that are encoded by three genes located on different chromosomes. However, in flies, there is only one miR-9 gene. In mouse embryos at embryonic day 10.5 (E10.5), pre-miR-9-2 is expressed at a much higher level than pre-miR-9-3, and pre-miR-9-1 expression is barely detectable [39]. Similarly, pre-miR-9-2 is expressed at high levels in human neural progenitor cells (hNPCs) derived from human embryonic stem cells (hESCs), while pre-miR-9-1 is almost undetectable [40]. In the developing mouse brain or zebrafish nervous system, miR-9 is also encoded by multiple genes and is broadly expressed, mostly in proliferating progenitor cells but it is also detectable in differentiated neurons [41-44]. It remains to be determined whether different miR-9 precursors may be expressed through distinct transcriptional controls in different subset of cells or at slightly different developmental stages. If that is the case, the presence of multiple genes encoding the same mature miRNA may confer another layer of regulation.

miR-124 (also known as miR-124a) was first identified as one of the mouse brain-specific miRNAs [34], and its nucleotide sequence is conserved from *Aplysia*, *Drosophila*, and *C. elegans *to mammals [35,37,45,46]. It is the most abundant miRNA in the brain, where it accounts for an estimated 25% to 48% of all miRNAs [34]. miR-124 is upregulated during neuronal differentiation of certain cell lines and hESCs and during mouse embryonic brain development [35,36,40,47]. miR-124 is widely expressed in virtually all postmitotic neurons in the adult mouse brain, but its expression is relatively low in the ventricular zones in the embryonic mouse brain [41]. Similarly, miR-124 is expressed in all differentiating cells throughout the larval zebrafish brain and retina [42] and in all differentiating and mature neurons in chick spinal cord [48,49]. Interestingly, in *Aplysia*, miR-124 is expressed at a high level in sensory neurons but is almost undetectable in motor neurons [45], suggesting functional divergence of this miRNA in different species. Like miR-9, miR-124 is encoded by one gene in some other model organisms but by three genes located on three different chromosomes in mammals. Although, like many other miRNAs, the nucleotide sequence of miR-124 precursors (pre-miR-124) is also poorly conserved in different species, they all maintain the stem-loop structures that produce the highly conserved mature miR-124.

## miR-9 in early neurogenesis

**Figure 1:**
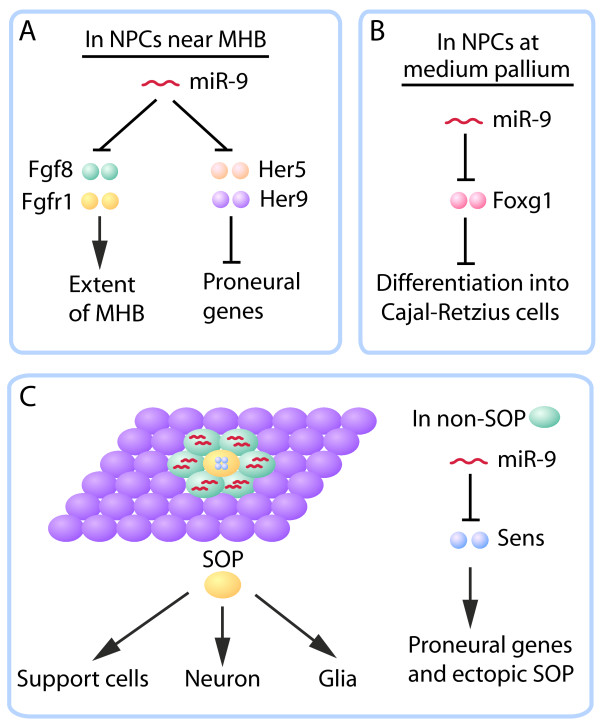
**Context-dependent functions of miR-9 in neurogenesis**. **(A,B) **In the developing brains of zebrafish (A) and mice (B), miR-9 is expressed in neural progenitor cells (NPCs) and promotes neurogenesis by downregulating different suppressors of neuronal differentiation. **(C) **During early neurogenesis in *Drosophila *embryos, miR-9 is not expressed in sensory organ precursors (SOPs) that eventually give rise to sensory neurons and other cell types. Instead, it is expressed in non-SOP cells, including those adjacent to the SOP in the pro-neural cluster, to suppress the residual expression of Sens, an activator of proneural genes in the process of lateral inhibition. Fgf, fibroblast growth factor; Fgfr, fibroblast growth factor receptor; Foxg1, forkhead box protein G1; MHB, midbrain-hindbrain boundary.

Detailed *in situ *hybridization reveals a dynamic expression profile for miR-9 during mouse corticogenesis. One of the most striking features is the reciprocal gradient of miR-9 and forkhead box protein G1 (*foxg1*) mRNA expression in E12 developing telencephalon [39]. Foxg1, a transcription factor that promotes the proliferation of cortical progenitor cells [50], is present throughout the telencephalon, but its expression gradually decreases in the medial pallium, where miR-9 is intensely expressed, raising the possibility that miR-9 may negatively regulate *foxg1 *expression [39]. Indeed, the *foxg1 *3' UTR contains an evolutionarily conserved miR-9 binding site and seems to be a direct target of miR-9. In P19-derived cells or in E12.5 neocortex, miR-9 knockdown increases Foxg1 expression, while overexpression of miR-9 decreases the protein levels of Foxg1 but not Nr2E1 (Nuclear receptor subfamily 2, group E member 1; also known as the human homologue of the *Drosophila *tailless gene (TLX)). Such a miRNA-target interaction supports the notion that miR-9 promotes the generation of Cajal-Retzius cells in the medial pallium of developing telencephalon [39] (Figure 1A). It will be interesting to confirm such a regulatory role for miR-9 in this developmental process *in vivo *using genetic approaches.

In zebrafish, miR-9 seems to affect early brain patterning through a different set of targets. Loss of both maternal and zygotic Dicer in zebrafish does not affect axis formation and differentiation of different cell types but causes abnormal morphogenesis of the developing brain [51], suggesting individual miRNAs may play fine-tuning functions. Indeed, miR-9 is widely expressed in neural progenitor cells in the developing zebrafish neural tube but is absent at the midbrain-hindbrain boundary (MHB) [44], an organizing center to specify the tectum at its rostral side and the cerebellum at its caudal side [52]. miR-9 seems to simultaneously target several components in the fibroblast growth factor signaling pathway, which is highly active in the MHB and restricts its patterning activity [44] (Figure 1B). miR-9 also regulates the expression of Her5 and Her9 during neuronal differentiation [44]. Both loss- and gain-of-function studies reveal that miR-9 restricts the organizing activity of the MHB and promotes neurogenesis in the midbrain-hindbrain region near the MHB.

The role of miR-9 in early neurogenesis is drastically different in *Drosophila *(where the gene is called *miR-9a*). Although the miR-9 nucleotide sequence is 100% conserved among many species, miR-9a shows little expression in the nervous system of developing *Drosophila *embryos; rather, it is highly expressed in ectodermal epithelial cells and in wing disc cells but not in sensory organ precursor (SOP) cells [37,38]. Thus, transcriptional regulation of *miR-9 *expression is not evolutionarily conserved. SOPs, which give rise to sensory neurons and supporting glial cells, are generated through a process called lateral inhibition, which involves the Notch signaling pathway and has been used as a model system for studying early neurogenesis [53]. Loss of miR-9a does not affect the viability of the mutant flies but increases the production of SOPs [38] (Figure 1C). The effects of miR-9 on SOP specification are not highly penetrant, again supporting the notion that many miRNAs are not absolute developmental switches. In flies, unlike in vertebrates, key targets of miR-9 are dLMO (*Drosophila *LIM only protein) [54,55] and Senseless (*sens*) [38,55] (Figure 1C), a zinc finger transcription factor downstream of Notch [56]. Since miR-9 binding sites in the *sens *3' UTR are not conserved in mammals, the shift in miR-9 targets may explain in part its diverse functions in different model organisms [57].

## miR-9 in stem cell-derived neural progenitor cells

miR-9 is upregulated during *in vitro *neural differentiation of mouse ESCs [58] and adult neural stem/progenitor cells [59], and during the maturation of hNPCs derived from hESCs [40]. Thus, miR-9 is expected to modulate the cellular behavior of stem cell-derived NPCs. Indeed, manipulation of miR-9 activity in mouse ESCs *in vitro *affects the ratio of differentiated neurons versus glia cells [58]. Similarly, overexpression of miR-9 in adult NPCs promotes neuronal differentiation and migration. However, inhibition of miR-9 activity does not affect the neuronal differentiation of adult NPCs [59], even though it impairs the generation of Cajal-Retzius neurons in embryonic mouse brains [39]. This discrepancy could be explained by the difference in the cellular context or some other unknown reasons. One target that mediates the effects of miR-9 overexpression on adult NPCs is TLX, a nuclear receptor required to maintain self-renewal of adult NPCs [59]. Interestingly, the transcription of pri-miR-9-1 also seems to be regulated by TLX, thus forming a potential feedback regulatory loop. However, if the relative levels of three pre-miR-9 genes in adult NPCs are similar to those in embryos, the change in total mature miR-9 level as regulated by this loop would be marginal because pre-miR-9-1 accounts for less than 5% of miR-9 precursors [39].

During neural differentiation of hESCs, miR-9 is not detectable in embryoid bodies and rosette structures; its expression is turned on at the onset of hNPC formation and increases gradually during hNPC maturation [40]. Inhibition of miR-9 activity in early hNPCs enhances migration and reduces proliferation without precocious differentiation. In this case, stathmin, which promotes microtubule instability [60], seems to be a key target required to mediate the effect of loss of miR-9. Partial suppression of stathmin by small interfering RNA rescues the effects of loss of miR-9 on the migration of early hNPCs *in vitro *and *in vivo *when transplanted into mouse embryonic brains or adult brains of a mouse model of stroke [40]. Thus, miR-9 may play distinct roles in NPCs of different developmental stages and origins.

## miR-124 in neuronal differentiation

The striking upregulation of miR-124 during neuronal differentiation [35,36] raises the possibility that this most abundant brain-specific miRNA may play unique functions during this process. Indeed, many targets of miR-124 that positively or negatively regulate neuronal differentiation have been identified. Ectopic expression of miR-124 in HeLa cells suppresses the expression of a large number of non-neuronal transcripts, leading to the hypothesis that one of miR-124's primary functions is to maintain neuronal identity by downregulating non-neuronal mRNAs [61]. Consistent with this notion, some of these targets are upregulated in postmitotic rodent neurons when miR-124 is knocked down, and miR-124 expression in non-neuronal cells and neural progenitor cells is suppressed by the RE1 silencing transcription factor (REST) [47]. Similarly, miR-124 directly targets the mRNA of polypyrimidine tract-binding protein 1 (PTBP1), a global repressor of alternative splicing in non-neuronal cells, leading to a more neuron-specific alternative splicing pattern [62]. In chick spinal cord, the mRNA of small C-terminal domain phosphatase 1 (SCP1) seems to be complementary to that of miR-124 in the developing spinal cord [49]. miR-124 also downregulates other endogenous targets during neuronal differentiation, such as laminin γ1 and integrin β1 in developing chick spinal cord [48] and ephrin-B1 in developing mouse cortex [63]. In the subventricular zone of the adult mouse brain, miR-124 is upregulated during the transition from transit-amplifying cell to neuroblasts, and its expression in neuroblasts increases further at cell cycle exit [64]. During this process, the high mobility group box transcription factor Sox9 seems to be a key target of miR-124 [64]. Evidently, miR-124 regulates different targets during neuronal differentiation in a cellular context-dependent manner.

Several miR-124-target interactions have been well established, but their relevance to a discernable developmental phenotype is less clear. miR-124 promotes neuronal differentiation in developing chick spinal cord, as shown by overexpression or 2'-OMe antisense knockdown experiments [49]. However, a similar study using the same assay system did not observe such an effect [48]. Although several reports indicate that ectopic overexpression of miR-124 promotes neuronal differentiation from progenitor cells [49,58,62-65], the precise roles of endogenous miR-124 in this developmental process remain to be further elucidated. *In vitro *acute knockdown of miR-124 in *ephrin-B1 *(*EfnB1*)^-/- ^NPCs modestly inhibited their neuronal differentiation [63]. *In vivo *knockdown of miR-124 in the subventricular zone of adult mice decreased the number of newly generated postmitotic neurons by 30% [64], suggesting an instructive role for miR-124 in promoting adult neurogenesis. In contrast, genetic ablation of miR-124 in *C. elegans *altered gene expression but did not result in any obvious defects in sensory neuron differentiation [66]. More sensitive assays and readouts are needed to further understand the subtle but apparently important functions of miR-124 in neuronal differentiation, especially using loss-of-function mutants in different model organisms.

## miR-9 and miR-124 in dendritic branching

Conditional knockout of Dicer in excitatory forebrain neurons in mice reduces dendritic branch elaboration [67]. In *Drosophila*, terminal dendritic branches of *Dicer-1 *mutant sensory neurons exhibit growth defects [68], and loss of Dicer-1 or Pasha in *Drosophila *olfactory projection neurons leads to a specific dendritic targeting defect [69]. Although Dicer may process other classes of RNAs, these findings raise the possibility that at least some miRNAs participate in the molecular regulation of dendritic morphogenesis. Indeed, both loss- and gain-of function studies of cultured developing cortical or hippocampal neurons indicate a role for miR-132 in basal and activity-dependent dendritic growth and branching [28,70]. As the most abundant brain miRNA whose expression persists throughout adult life, miR-124 seems to promote neurite outgrowth in differentiating mouse P19 cells, possibly in part by regulating members of the Rho GTPase family [71]. However, ectopic expression of miR-132 or miR-124 had no effect on dendritic growth or arborization of hippocampal neurons that had been cultured *in vitro *for 14 days [72]. The latter result could be explained by the high levels of these miRNAs already present in mature neurons in culture. The involvement of the miR-124-target interaction in dendritic morphogenesis is further revealed by manipulating the 3' UTR of BAF53b, a key component of the ATP-dependent chromatin-remodeling complexes [73]. Loss of the miR-124 and miR-9* binding sites in the BAF53a 3' UTR inhibited activity-dependent dendritic growth in cultured hippocampal neurons, while expression of BAF53b with the wild-type BAF53a 3' UTR failed to produce such an inhibition [73]. Thus, miR-124 downregulates BAF53a, which in turn leads to increased activity-dependent dendritic growth.

Ectopic expression of miR-124 in developing *Drosophila *sensory neurons suppresses dendritic branching [68]. The different effects of miR-124 in P19 cells versus fly neurons may reflect the difference in mRNA targets in different cell types. However, the precise roles of endogenous miR-124 in dendritic development await further investigation once miR-124 mutant flies or knockout mice become available. In contrast to miR-124, ectopic expression of miR-9 in fly sensory neurons increases dendritic branching [68], suggesting that different miRNAs can exert opposite effects on this developmental process through distinct subsets of target mRNAs. Whether endogenous miR-9 in mammalian neurons also regulates dendritic morphogenesis remains to be seen.

## miR-9 and miR-124 in synaptic plasticity and brain function

Synaptic formation and plasticity play central roles in neuronal connectivity and brain function, and miRNAs seem to be well positioned to regulate this important process [74]. Indeed, loss of Dicer *in vivo *not only reduces dendritic branching but also affects spine morphology [67], although the interpretation of this result is complicated by the cell death phenotype caused by conditional loss of Dicer in certain neurons [67,75,76]. Moreover, several miRNAs have been implicated in spine morphogenesis and synaptic plasticity in *C. elegans*, *Drosophila*, and mammals, including miR-134 [24], let-7 [77,78], miR-284 [79], miR-1 [80], miR-138 [31], miR-206 [81], and miR-125a [72].

This rapidly expanding list also includes miR-124, which in *Aplysia*, in stark contrast to that in other model organisms, does not seem to be expressed ubiquitously and constitutively in all neurons [45]. In *Aplysia *sensory-motor neuron co-culture, a model system for studying short- and long-term memory [82], miR-124 is rapidly downregulated by the neurotransmitter serotonin. This downregulation is relevant to synaptic plasticity because manipulating miR-124 levels in sensory neurons directly affects long-term facilitation at the sensory-motor synapse [45]. One of the predicted mRNA targets of miR-124 is CREB1, a transcriptional activator required for long-term facilitation [83]. Indeed, the expression of *Aplysia *CREB1 is directly inhibited by miR-124, and miR-124 suppresses serotonin-induced synaptic facilitation through downregulation of CREB1 [45]. The miR-124 binding site is conserved in the mammalian CREB1 3' UTR. Whether CREB-mediated signaling and synaptic functions are regulated by  miR-124 in the mouse brain remains to be experimentally validated. Interestingly, miR-124 and other neuronal miRNAs have a much shorter half-life than that in non-neuronal cells and their abundance in mammalian neurons is regulated by neuronal activity [84]. Further investigation of the underlying mechanism will be of great importance.

In the adult rat brain, miR-124 is significantly downregulated after cocaine administration, suggesting that this miRNA may be involved in cocaine-induced plasticity, possibly through CREB, brain-derived neurotrophic factor (BDNF), or other potential targets [85]. Similarly, miR-9 is expressed in supraoptic nucleus neurons and striatal neurons in the rat brain, as detected by single-cell PCR, and alcohol increases miR-9 expression in both of these cell types [86]. miR-9 downregulates specific mRNA splice variants of the large conductance calcium- and voltage-activated potassium (BK) channel, contributing to the development of alcohol tolerance [86]. Thus, the BK channel is a key target of miR-9 in drug adaptation and adult brain plasticity.

The potential involvement of miRNAs in age-dependent neurodegeneration is increasingly appreciated [18]. For instance, several miRNAs suppress the neurotoxicity of atrophin 1 in spinocerebellar ataxia 1 (SCA1) pathogenesis in a combinatorial manner [87]. miR-206 plays an active role in delaying the disease progress of amyotrophic lateral sclerosis [81], a fatal disease caused by motor neuron degeneration in which dysregulation of the miRNA pathway may be one of the most significant pathogenic mechanisms [88]. Interestingly, miR-9 is significantly reduced in a genetic model of spinal motor neuron disease [89]. Similarly, miR-9 levels are lower in patient brains affected by Huntington's disease [90], and miR-29a/b-1 expression is reduced in the brains of patients with sporadic Alzheimer's disease [91]. Whether these brain-specific miRNAs contribute to the pathogenesis of some age-dependent neurodegenerative diseases remain to be further investigated.

## Conclusions

Although a few miRNAs can function as developmental 'switches' similar to transcription factors to fundamentally affect cell fate, such as in the specification of chemosensory neurons in *C. elegans *[19] and some aspects of cardiovascular development [92], many other miRNAs, such as miR-9 and miR-124, individually exert a more modest effect on neuronal development. Another similarity between the two most extensively studied neuronal miRNAs is their modest effects on gene expression, consistent with recent reports that many if not all miRNAs mostly induce less than twofold changes in target gene expression [93,94]. Thus, these miRNAs may serve as an important buffering system to ensure the precision of gene regulation and tissue homeostasis in developing and adult brains.

Both miR-9 and miR-124 are implicated in multiple stages of neuronal development. It is intriguing that, in some instances, miR-124 and miR-9 are needed to act cooperatively with each other [58,73] and as parts of regulatory feedback loops involving REST [47,90]. Although these regulatory networks can be quite complicated with multiple transcription factors and miRNAs involved, a recurring theme seems to be that one or a few mRNA targets account for the majority of the phenotype in a particular developmental or cellular process (Tables 1 and 2). This is likely the case for many other miRNAs as well. The context-dependent functions of miRNAs in neuronal development or other processes could be explained in part by the variations in transcriptome composition in diverse cell types in different species. The ratio of copy numbers between a specific miRNA and its target may also influence its developmental functions. Thus, it will be useful to systematically identify context-dependent targets of a specific miRNA, such as using an *in vivo *crosslinking and immunoprecipitation (CLIP) approach [95,96]. Moreover, it is critically important to study the endogenous activities of specific miRNAs in their physiological contexts, and results obtained from heterologous assay systems need to be interpreted with sufficient caution.

**Table 1:** mRNA targets and functions of miR-9 in neuronal development and function

Functions	Species	Targets	References
Suppresses excess SOP production	*D. melanogaster*	Sens	[38,55]
Promotes dendritic branching	*D. melanogaster*	?	[68]
Restricts the extent of MHB	Zebrafish	FGF8, FGFR1	[44]
Promotes neuronal differentiation near MHB	Zebrafish	Her5, Her9	[44]
Limits the generation of Cajal-Retzius cells	Rodent	Foxg1	[39]
Promotes neuronal differentiation from adult neural stem/progenitor cells	Rodent	TLX	[59]
Enhances alcohol tolerance in adult brains	Rodent	BK channels	[86]
Inhibits astroglial cell differentiation	Rodent	?	[58]
Promotes proliferation but limits migration of hESC-derived young hNPCs	Human	Stathmin	[40]
May contribute to neurodegenerative diseases	Human	NEFH, REST	[89,90]

BK channel, large conductance calcium- and voltage-activated potassium channel; FGF, fibroblast growth factor; FGFR, fibroblast growth factor receptor; Foxg1, forkhead box protein G1; hESC, human embryonic stem cell; hNPC, human neural progenitor cell; MHB, midbrain-hindbrain boundary; NEFH (neurofilament heavy polypeptide); REST, RE1 silencing transcription factor; SOP, sensory organ precursor; TLX, human homologue of the *Drosophila *tailless gene.

**Table 2:** mRNA targets and functions of miR-124 in neuronal development and function

Functions	Species	Targets	References
Inhibits long-term facilitation at the sensory-motor synapses	*Aplysia*	CREB	[45]
Suppresses dendritic branching by overexpression	*D. melanogaster*	?	[68]
Promotes neuronal differentiation in spinal cord	Chick	SCP1	[49]
Promotes neurite growth	P19 cells	RhoA	[71]
Involved in BAF53b-induced activity-dependent dendritic growth	Rodent	BAF53b	[73]
Promotes neuronal differentiation in developing brain	Rodent	REST, PTBP1, Ephrin-B1	[62-64]
Promotes adult neurogenesis	Rodent	Sox9	[65]
Promotes neuronal differentiation	Rodent	?	[58]
Cocaine-induced plasticity in the adult brain	Rodent	CREB, BDNF	[85]

BDNF, brain-derived neurotrophic factor; CREB, cAMP response element binding protein; PTBP1, polypyrimidine tract-binding protein 1; REST, RE1 silencing transcription factor; SCP1, small C-terminal domain phosphatase 1.

Although only a limited number of miRNAs have been studied for their endogenous functions in the nervous system, the importance of this class of regulatory molecules in the construction of neuronal circuits is becoming increasingly evident. Intriguingly, despite evolutionary conservation at the nucleotide level, the expression patterns and regulatory targets of many miRNAs shifted during evolution. miR-9 and miR-124 are among the most ancient animal miRNAs that show cell-type specific expression and may play key roles in the development of new body plans [97]. Thus, conserved neuronal miRNAs may assume novel functions, which, together with newly evolved miRNAs, such as those uniquely expressed in the human brain [98], may contribute to the evolution of this most complex yet poorly understood organ.

## Abbreviations

BK: channel, large conductance calcium- and voltage-activated potassium channel; CREB: cAMP response element binding protein; E: embryonic day; Foxg1: forkhead box protein G1; hESC: human embryonic stem cell; hNPC: human neural progenitor cell; MHB: midbrain-hindbrain boundary; miRNA: microRNA; pri-miRNA: primary microRNA; REST: RE1 silencing transcription factor; SOP: sensory organ precursor; TLX: human homologue of the *Drosophila *tailless gene; UTR: untranslated region.

## Competing interests

The author declares he has no competing interests.
